# Acute Myeloid Leukemia Cells Express ICOS Ligand to Promote the Expansion of Regulatory T Cells

**DOI:** 10.3389/fimmu.2018.02227

**Published:** 2018-09-26

**Authors:** Yixiang Han, Yuqing Dong, Qianqian Yang, Wanling Xu, Songfu Jiang, Zhijie Yu, Kang Yu, Shenghui Zhang

**Affiliations:** ^1^Central Laboratory, The First Affiliated Hospital of Wenzhou Medical University, Wenzhou, China; ^2^Department of Hematology, Wenzhou Key Laboratory of Hematology, The First Affiliated Hospital of Wenzhou Medical University, Wenzhou, China; ^3^Division of Clinical Research, The First Affiliated Hospital of Wenzhou Medical University, Wenzhou, China

**Keywords:** acute myeloid leukemia, regulatory T cells, inducible T-cell costimulator ligand, inducible T-cell costimulator, interleukin-10

## Abstract

CD4^+^CD25^+^Foxp3^+^ regulatory T cells (Tregs) accumulate in bone marrow microenvironment in acute myeloid leukemia (AML). However, little is known about how the tumor environment including tumor cells themselves affects this process. Here we demonstrated that AML cells expressed inducible T-cell costimulator ligand (ICOSL) that can provide costimulation through ICOS for the conversion and expansion of Tregs sustaining high Foxp3 and CD25 expression as well as a suppressive function. TNF-a stimulation up-regulated the expression of ICOSL. Furthermore, both the conversion and expansion of CD4^+^CD25^+^Foxp3^+^ T cells and CD4^+^ICOS^+^Foxp3^+^ T cells were induced by co-culture with AML cells overexpressed ICOSL. CD4^+^CD25^+^ICOS^+^ T cells possessed stronger ability to secrete IL-10 than CD4^+^CD25^+^ICOS^−^ T cells. The mechanism by which IL-10 promoted the proliferation of AML cells was dependent on the activation of the Akt, Erk1/2, p38, and Stat3 signaling pathways. Blockade of ICOS signaling using anti-ICOSL antibody impaired the generation of Tregs and retarded the progression of an AML mice model injected with C1498 cells. The expression of ICOSL of patient AML cells and ICOS^+^ Tregs were found to be predictors for overall survival and disease-free survival in patients with AML, with ICOS^+^ Treg cell subset being a stronger predictor than total Tregs. These results suggest that ICOSL expression by AML cells may directly drive Treg expansion as a mechanism of immune evasion and ICOS^+^ Treg cell frequency is a better prognostic predictor in patients with AML.

## Introduction

Acute myeloid leukemia (AML) is a genetically complex and heterogeneous set of diseases characterized by overwhelming accumulation of immature myeloid cells in the bone marrow and peripheral blood. Despite considerable advances in understanding of the molecular basis of AML pathogenesis, overall survival in adult patients with AML has improved only modestly in the past 3 decades (1).

Besides genetic abnormalities of malignant cells themselves, the aberrant status of tumor microenvironment plays a crucial role on disease progression (2). As an important component of microenvironment, regulatory T cells (Tregs), characterized by their expression of the transcription factor Foxp3, are required to maintain immune homeostasis and prevent excessive tissue damage (3). Tregs comprise natural and inducible Tregs, and usually express CD25, glucocorticoid-induced TNFR-related protein (GITR), cytotoxic T lymphocyte associated antigen 4 (CTLA-4) and CD45RO, and lack CD127 (3). Tregs can suppress the proliferation and cytokine secretion of responding effector T cells (Teffs) to prevent autoimmune diseases, allergies, infection-induced organ pathology as well as transplant rejection (4, 5). However, Tregs can be deleterious in cancer through suppression of anti-tumor immunity (6). It has been found that Tregs actively accumulate in distinct tumor microenvironments, and their presence associates with poor antitumor immune response and poor survival (7, 8). In AML, we have demonstrated that 11.9% of the CD4^+^ T cells found in bone marrow are CD25^+^CD127^lo^, with only 9.19% found in the peripheral blood of the same patients (9). These results indicate that AML cells attract and retain Tregs in the bone marrow, or that the tumor microenvironment can promote the expansion of tumor-infiltrating Tregs. Additionally, it has been reported that the tumor microenvironment can favor the conversion of inducible Tregs from CD4^+^CD25^−^T cells (10), with the total pool of Tregs being comprised of both natural Tregs and induced peripheral Tregs.

The development of Tregs is dependent on intracellular signal transduction events responded to stimulation by the T cell receptor (TCR), co-stimulatory molecules, and cytokines. Inducible T-cell costimulator (ICOS), being a member of the CD28 family of co-stimulatory molecules, is implicated in maintaining durable immune reactions upon binding to ICOS ligand (ICOSL). ICOS costimulation of CD4^+^ T cells favors the production of Th2 cytokines such as IL-4, IL-10, and IL-13 (11). Particularly, ICOS/ICOSL axis plays a crucial role in Treg cell function and promotes Treg differentiation through activating the phosphoinositide 3-kinase/Akt pathway (12). The finding that H.polyguras elicited Foxp3^+^ T cells were all negative for expression of Helios, a specific marker of thymic-derived Treg cells marker (13), and this population was absent in ICOS^−/−^ mice, suggests that ICOS affects the induction of Treg cells predominantly in the periphery (14). Recent studies showed that follicular lymphoma cells generate Treg cells via ICOS/ICOSL cascade and are susceptible to treatment by anti-ICOS/ICOSL therapy (15). Accumulating evidence has demonstrated that multiple normal tissues express ICOSL and regulate CD4^+^ T-cell activation and cytokine production (12, 16). Tregs present in tumor tissues have been found to express ICOS on their surface, and ICOS^+^ Tregs have been demonstrated to powerfully dampen T-cell responses indirectly through impairing antigen-presenting cells (APCs) with interleukin (IL)-10 (17). The source of ICOS costimulation for Tregs still remains largely unknown. In AML, it has been reported that AML cells express ICOSL (18, 19), whereas how ICOSL expressed by AML cells regulate CD4^+^ T-cell activation has not been characterized yet.

In this study, we reported that multiple AML cell lines and primary AML cells expressed ICOSL on their surface and could co-stimulate Tregs to promote high expression of CD25, Foxp3, and ICOS. We also observed an increased frequency of ICOS^+^ Tregs secreted IL-10 as a positive feedback to promote the proliferation of AML cells. Anti-ICOSL antibody inhibited the generation of ICOS^+^ Tregs and retarded the progression of AML in a murine model. ICOS^+^ Treg cell frequency may be a better predictor than total Treg cell frequency in patients with AML.

## Materials and methods

### Selection of patients

This study was approved by the Institutional Ethics Committee of the First Affiliated Hospital of Wenzhou Medical University, and informed consent was obtained from all participants in accordance with the Declaration of Helsinki. A total of 121 newly diagnosed adult patients with AML excluding APL with t (15, 17) (q22;q12); PML-RARA consisted of 69 males and 52 females with a mean age of 40 years old (range: 17–60) were enrolled at Department of Hematology of the First Affiliated Hospital of Wenzhou Medical University (Table S1). The diagnosis and subtype identification of AML were established according to the 2008 WHO Classification for AML (20). The age-matched control group consisted of 40 healthy donors, which included 17 males and 23 females with a mean age of 41 years old (range: 19–62). All peripheral blood and bone marrow mononuclear cells (BMMNCS) were isolated using Ficoll-hypaque density gradient centrifugation and stored, and meanwhile, peripheral blood plasma and bone marrow plasma were also obtained and stored.

### Isolation and culture of TREGs and TEFFs

ICOS^+^CD4^+^CD25^high^ T cells, ICOS^−^CD4^+^CD25^high^ T cells, and CD4^+^CD25^−^ effector T cells (Teffs) were isolated from BMMNCs of 5 patients with AML by FACSAria III (Becton Dickinson, San Jose, CA, USA). The suppressive function of ICOS^+^CD4^+^CD25^high^ T cells and ICOS^−^CD4^+^CD25^high^ T cells was determined using mixed leukocyte culture assay. Briefly, carboxyfluorescein diacetate succinimidylester (CFSE, 5 μmol/L)-labeled CD4^+^CD25^−^ T cells were incubated for 5 days with 20 μg/ml mitomycin-treated PBMCs in the presence of autogeneic ICOS^+^CD4^+^CD25^high^ T cells or ICOS^−^CD4^+^CD25^high^ T cells, with stimulation with plate-coated anti-CD3 (1 μg/ml) and soluble anti-CD28 (3 μg/ml) and IL-2 (20 ng/ml), and cell division was subsequently monitored by flow cytometric assay of CFSE dye dilution.

Purified CD4^+^ T cells, isolated from PBMNCs of healthy donors using MACS CD4^+^ T cell isolation kit (Miltenyi Biotec, Bergisch Gladbach, Germany), were seeded at 5 × 10^4^ cells/well on 96-well plates coated with 1 μg/ml anti-CD3 monoclonal antibody (eBiosciences, San Diego, CA, USA) and stimulated with 3 μg/ml anti-CD28 monoclonal antibody (eBiosciences) and 20 ng/ml IL-2 for 5 days. Full-length hICOSL cDNA was cloned into eukaryotic expression vector GV230. HEL or HL-60 cells were transfected transiently with the positive clones of constructed recombinant plasmid GV230-hICOSL, and were subsequently screened with 800 μg/ml G418 for 7 days. QPCR and western blot were performed to determine the mRNA and protein levels of ICOSL to confirm the overexpression of ICOSL. After treatment with 20 μg/ml mitomycin for 1 h, AML cells or AML overexpressed ICOSL were co-cultured with CD4^+^ T cells at a ratio of 1:1 for 5 days in the presence or absence of 10 μg/mL neutralizing anti-ICOSL antibody (eBiosciences), with stimulation with plate-coated anti-CD3 (1 μg/ml) and soluble anti-CD28 (3 μg/ml) and IL-2 (20 ng/ml).

### Immunostaining and flow cytometric analysis

The surface and intracellular immunostaining for flow cytometric analysis was performed as described previously (9). Briefly, the cells were treated with human immunoglobulin to block non-specific binding and then stained with appropriate fluorochrome-conjugated surface monoclonal antibodies (mAbs), including anti-ICOS-PE, anti-CD4-PerCP, and anti-CD25-APC (all from BD Pharmingen, San Diego, CA, USA), followed by incubating in the dark for 30 min at 4°C. Isotype-matched negative controls were used in all assays. For intracellular Foxp3 staining, the cells were stained with appropriate surface mAbs, and subsequently fixed, permeabilized and incubated with anti-Foxp3-PE (BD Pharmingen) for 30 min at 4°C. For analysis of intracellular cytokine staining, PBMCs, Tregs or Teffs were stimulated for 5 h with phorbol 12-myristate 13-acetate (PMA) and ionomycin in the presence of brefeldin A (all from Sigma-aldrich, St.Louis, MO, USA). After staining with surface mAbs, the cells were fixed, permeabilized, and stained with mAbs against cytokines including IFN-γ, IL-2, IL-17, and IL-4 (all from BD Pharmingen). Acquisition and analysis were performed on a flow cytometry (FACSCalibur; Becton Dickinson). AML blasts were gated on CD45^dim^SSC^dim^ and the expression of ICOSL was assessed based on the percentage of positive cells.

### Quantitative RT-PCR for gene expression analysis

CD33^+^CD45^dim^ cells were sorted from BMMNCs of healthy donors and patients with AML using FACSAria III, respecitively. Total RNA was extracted using Trizol (Invitrogen, Carlsbad, CA, USA) and reverse transcribed into cDNA, and then cDNA was amplified and quantified on an ABI Prism 7500 Sequence Detection System (Applied Biosystems, Foster city, CA, USA) using a SYBR Green PCR master mix (Takara, Dalian, China) with primer pairs (Table S2), respectively.

### Cytokine analysis

The plasma samples were collected from peripheral blood and bone marrow of healthy donors and newly diagnosed patients with AML. The CD4^+^CD25^high^ICOS^+^ cells and CD4^+^CD25^high^ICOS^−^ cells were sorted and cultured in 200 μl X-VIVO™ 15 (Lonza, Walkersville, MD, USA) supplemented with 20 ng/ml IL-2. After 2 days, the supernatants were collected and the levels of IL-10 were measured using a commercial ELISA kits from MultiSciences (Hanzhou, China) according to the manufacturer's protocols. Additionally, plasma levels of TNF-α were determined using an ELISA kits from Thermofisher Scientific (Vienna, Austria). Human AML cell lines HL-60, HEL, THP-1, or U937 as well as murine AML cell lines C1498 were treated with recombinant human or murine TNF-α 50 ng/ml (both from PeproTech, Rocky Hill, NJ, USA) for 48 h, and the expression of ICOSL was determines using immunostaining and flow cytometric analysis.

### Cell viability assay

HL-60 or HEL cells were seeded in 96-well plates at a density of 1 × 10^4^ cells/well and treated with various doses of IL-10 (PeproTech) for 24 h. Then, the cell viability was determined using the Cell Counting Kit-8 (CCK-8) assay according to the manufacturer's protocol (Dojindo, Kumamoto, Japan).

### Western blot assay

After treatment with 10 ng/ml IL-10 for different times, the cells were collected and lysed immediately using RIPA Lysis Buffer (Beyotime Institute of Biotechnology, Haimen, China) containing PMSF and Halt protease and phosphatase inhibitor cocktail (Pierce, Rockford, IL, USA). The protein was boiled and subjected to western blot with antibodies against Akt, p-Akt (Ser473), Erk1/2, p-Erk1/2 (Thr202/Tyr204), p38, p-p38 (Thr180/Tyr182), Stat3, p-Stat3 (Tyr705) or GAPDH (all from Cell Signaling Technology, Beverly, MA, USA), respectively.

### C1498 AML model

This study was carried out in accordance with the recommendations of “institutional guidelines, Wenzhou Medical University Animal Care and Use Committee.” The protocol was approved by the “Wenzhou Medical University Animal Care and Use Committee.” The C1498 AML model was established as described previously with minor modifications (21). Briefly, C57BL/6 mice (6–8 weeks old/20–25 g body weight) were purchased from Laboratory Animal Centre of Wenzhou Medical University. C1498 cells were cultured in complete DMEM supplemented with 10% fetal bovine serum. C1498-GFP cells were engineered by retroviral transduction using the pLEGFP plasmid. The GFP expression of C1498-GFP cells was maintained with puromycin (2 μg/ml) and periodically monitored by flow cytometry. Exponentially growing C1498 cells (5 × 10^6^) were suspended in 100 μl PBS, and then intravenously injected into the tail vein of recipient mice, which had been already exposed to 5 Gy myeloablative irradiation 4 h before. According to the method reported by Raynor et al. (22), these mice were injected intraperitoneally with 7.5 mg/kg anti-mouse ICOSL antibody (clone: HK5.3) or with rat IgG_2a_ isotype control (both from BioXcell, West Lebanon, NH, USA) on days 0, 3, 6, 9, 12, and sacrificed on day 15.

### Statistical analysis

The data were presented as mean ± SEM and analyzed by *t*-tests or one-way ANOVA followed by a *post hoc* Turkey's test to determine the differences between the groups. Differences were considered significant at *P* < 0.05.

## Results

### Expression and induction of ICOSL molecules on AML cells

**Figure 1:**
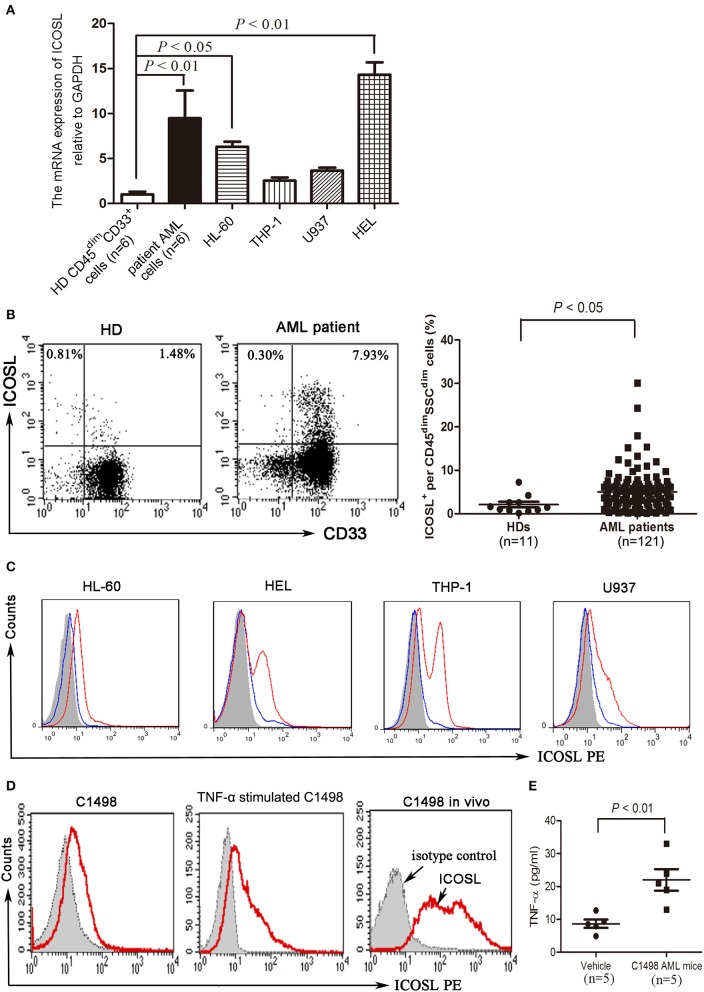
The expression of ICOSL in acute myeloid leukemia. **(A)** The mRNA expression of ICOSL in CD45^dim^CD33^+^ cells isolated from healthy donors, patient AML cells, and four AML cell lines HL-60, THP-1, U937, and HEL were determined and expressed as mean ± SEM representing at least three independent experiments. ANOVA was used to determine the differences between the groups. **(B)** Representative plots (left panel) and statistical data (right panel) showed that the expression of ICOSL in SSC^dim^CD45^dim^cells isolated from bone marrow of 11 healthy donors and 121 patients with AML. Unpaired *t*-test was used to determine the difference. **(C)** Treatment with TNF-a 50 ng/ml for 48 h induced the expression of ICOSL in HL-60, HEL, THP-1, and U937 cells. Overlay histograms showing antibody stains with (solid red) or without (solid blue) TNF-a stimulation and isotype stains (filled gray) are representatives of three independent experiments. **(D)** Treatment with murine TNF-α 50 ng/ml for 48 h induced the expression of ICOSL in C1498 cells. The expression of ICOSL was increased on day 5 when C1498-EGFP cells were injected *in vivo*. Overlay histograms showing antibody stains (solid red) and isotype stains (filled gray) are representatives of three independent experiments. **(E)** The plasma levels of TNF-α were determined by ELISA on day 5 in C57BL/6 mice intravenously injected with or without C1498 cells (5 × 10^6^/mouse). Unpaired *t*-test was used to determine the difference.

Compared with CD45^dim^CD33^+^ cells isolated from bone marrow of healthy donors, blasts cells from a substantial number of AML patients strongly expressed ICOSL at a transcriptional level (Figure 1A). Among the cell lines tested, HL-60 and HEL cells constitutively expressed ICOSL, and THP-1 and U937 cells expressed an increased but not statistically significant level of ICOSL mRNA (Figure 1A). The increased expression of ICOSL protein was also present on the plasma membrane of patient AML cells, consistent with the results of ICOSL mRNA (Figure 1B). However, the expression of ICOSL protein on the four AML cell lines tested was almost undetectable or very weak (Figure 1C). Since it has been well-established that TNF-α stimulation enhances the expression of ICOSL in several different cell types (23), we next determined whether TNF-α stimulation influences the expression of ICOSL on AML cells. TNF-α 50 ng/ml robustly up-regulated the expression of ICOSL in four AML cell lines tested (Figure 1C). Additionally, we also determined whether three other cytokines IFN-γ, IL-10, IL-17A or IL-21 affect the expression of ICOSL and found that these four cytokines did not change the expression of ICOSL on two AML cell lines HL-60 and HEL (Figure S1). The expression of ICOSL was very weak on the murine AML cell line C1498 and treatment with TNF-α 50 ng/ml for 48 h also induced the expression of ICOSL in C1498 cells *in vitro* (Figure 1D). Since it has been recognized that the level of TNF-α is elevated in AML patients (24, 25), we speculate that the expression of ICOSL on AML cells can be enhanced *in vivo*. As expected, the expression of ICOSL was increased when C1498 cells were injected intravenously into C57BL/6J mice (Figure 1D). Meanwhile, the mice injected C1498 cells had a higher plasma levels of TNF-α than the vehicle control mice (Figure 1E). The results suggested that AML cells express ICOSL and the expression of ICOSL is increased *in vivo* due to the stimulation of TNF-α.

### Bone marrow-infiltrating TREGs express high levels of ICOS

**Figure 2:**
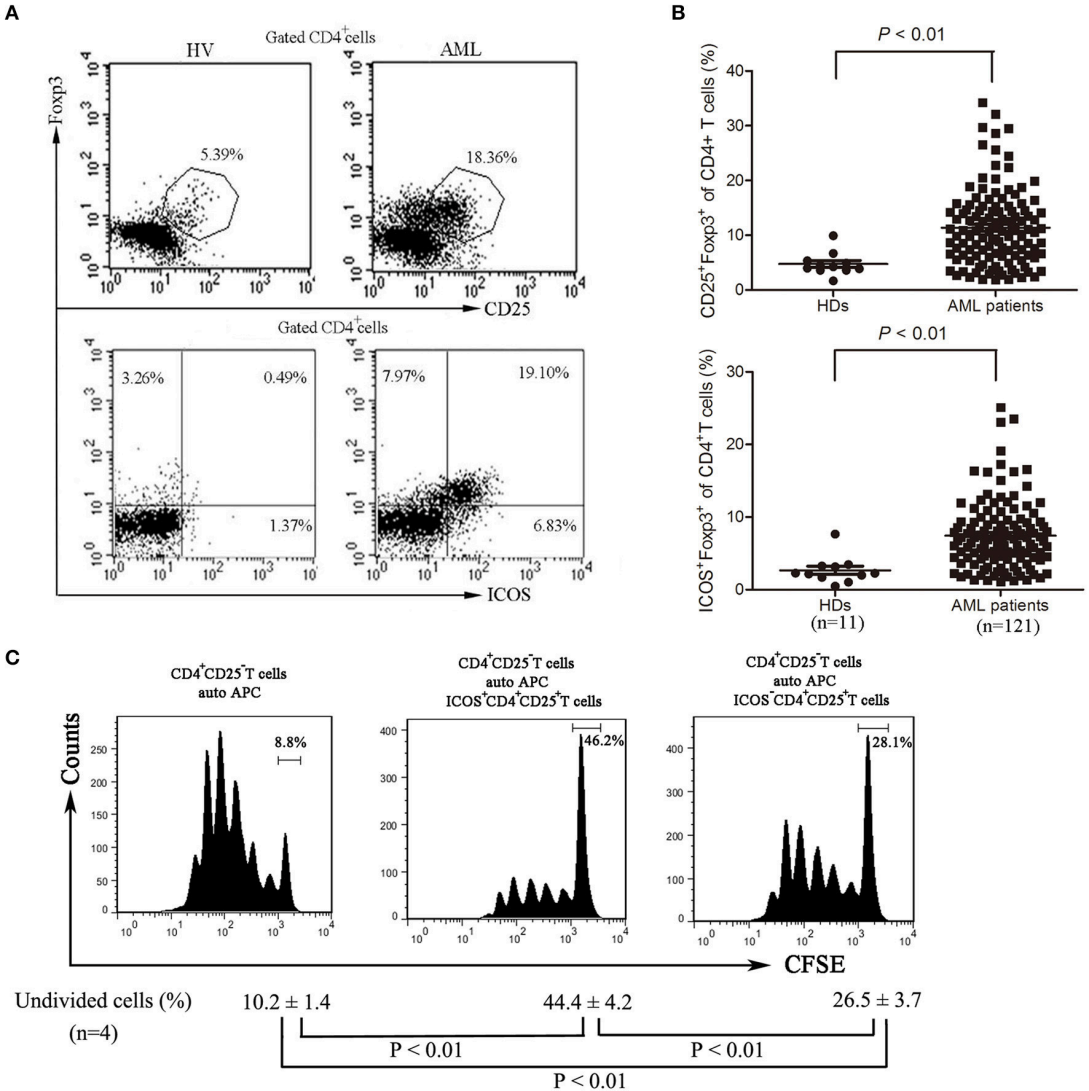
The frequencies and function of ICOS^+^ Tregs in patients with AML. **(A,B)** Representative plots (left panel) and statistical data (right panel) showed that the frequencies of CD4^+^CD25^+^FoxP3^+^ cells and CD4^+^FoxP3^+^ICOS^+^ cells in 11 healthy donors and 121 patients with AML. Unpaired *t*-test was used to determine the difference. **(C)** The CD4^+^CD25^high^ICOS^+^ cells and CD4^+^CD25^high^ICOS^−^ cells were sorted from bone marrow mononuclear cells of patients with AML using flow cytometry, and then incubated for 5 days with PBMCs treated with 20 μg/ml mitomycin and CFSE-labeled CD4^+^CD25^−^ T cells, with stimulation with plate-coated anti-CD3 (1 μg/ml) and soluble anti-CD28 (3 μg/ml) and IL-2 (20 ng/ml). The cell division was measured by levels of CFSE dilution by flow cytometry. Histograms were representatives of four independent experiments and ANOVA was used to determine the differences.

Although Tregs have been shown to be prevalent in the bone marrow microenvironment of AML patients (9), further characteristics of these cells still need to be well-identified. We analyzed the frequency of Treg cells and found that the frequency of Treg cells was significantly increased in bone marrow of AML patients compared with those of healthy donors (Figures 2A,B) as previously reported (9). Meanwhile, the expression of ICOS was also robustly increased in CD4^+^CD25^+^ Foxp3^+^ T cells (Tregs) from bone marrow of AML patients compared with those from healthy donors (Figures 2A,B). We next investigated the inhibitory capability of the ICOS^+^CD4^+^CD25^high^ T cells (ICOS^+^ Tregs) against the conventional effector T cells. As shown in Figure 2C, the proliferation inhibition of CFSE-labeled CD4^+^CD25^−^ T cells was greater using ICOS^+^ Tregs than ICOS^−^ Tregs from the same patients with AML.

### AML cells promotes the induction of TREG cells through the interaction of ICOS and ICOSL

**Figure 3:**
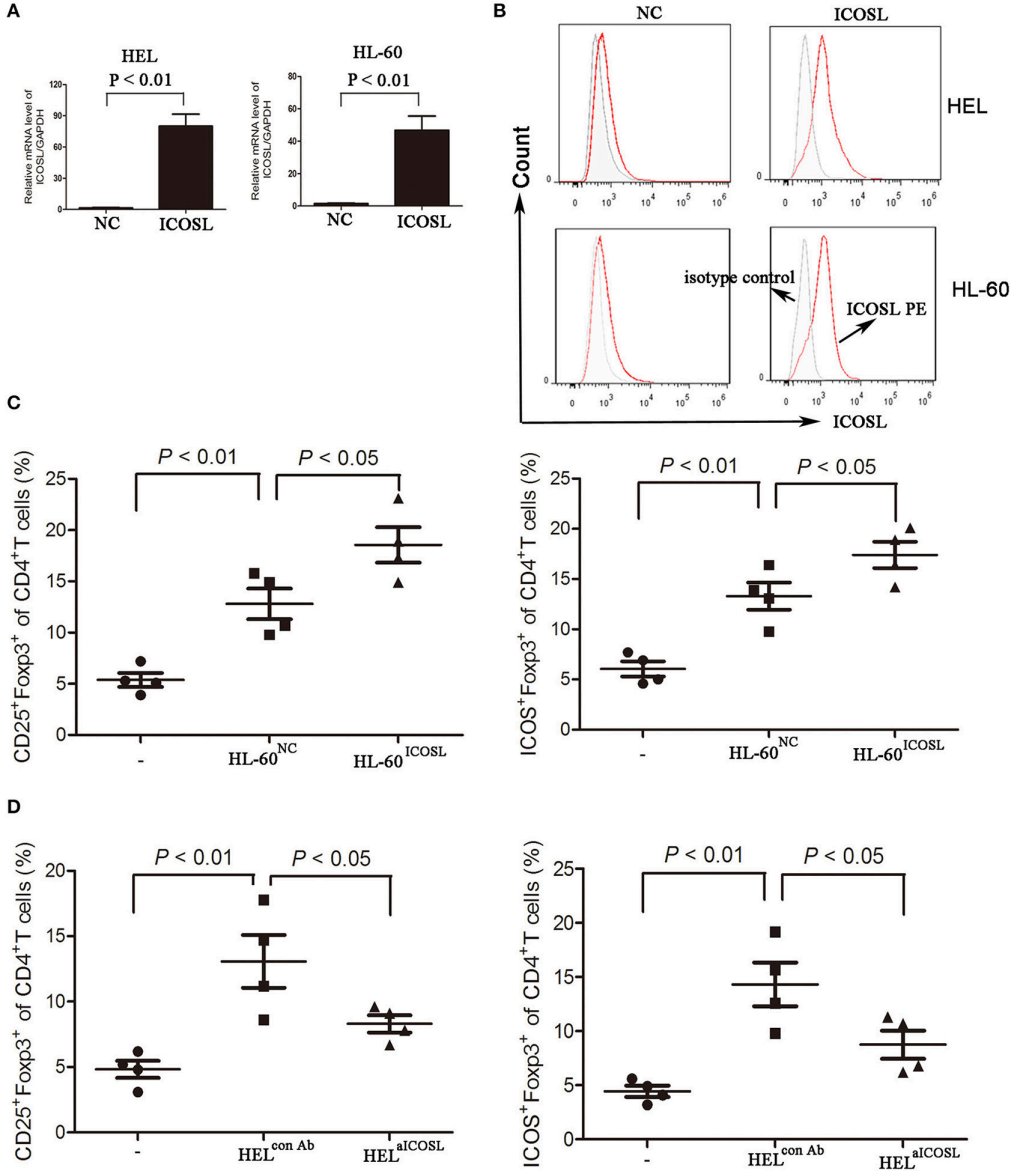
The effect of ICOSL in AML cells on Treg induction. **(A,B)** AML cell lines HL-60 and HEL were transduced to constitutively express full-length human ICOSL. Meanwhile, these two cell lines were transduced with the empty vector. The mRNA expression of ICOSL was determined and unpaired *t*-test was used to determine the difference. Overlay histograms showing cell surface protein expression of ICOSL with antibody stains (solid red) and isotype stains (filled gray) were representatives of three independent experiments. **(C)** CD4^+^ CD25^+^Foxp3^+^ cells and CD4^+^ICOS^+^Foxp3^+^ cells were significantly increased when CD4^+^ T cells co-cultured with HL-60 cells overexpressed ICOSL for 5 days. **(D)** Anti-ICOSL antibody reduced the expansion of CD4^+^CD25^+^Foxp3^+^ cells and CD4^+^ICOS^+^Foxp3^+^ cells in CD4^+^ T cells co-cultured with HEL cells for 5 days. Data were expressed as mean ± SEM representing four independent experiments and AVONA was used to determine the differences.

To evaluate the critical role of ICOSL as a putative Treg inducer, we overexpressed ICOSL in HEL cells and HL-60 cells by transducing them with plasmid carrying the full-length human ICOSL gene. ICOSL at both mRNA and cell surface protein levels were strongly expressed without any stimulators (Figures 3A,B). Under *in vitro* induction of Tregs, HL-60 overexpressed ICOSL induced more CD25^+^Foxp3^+^ Tregs from CD4^+^ T cells than those with HL-60 transduced with NC plasmids (Figure 3C). Meanwhile, Tregs induced by HL-60 cells overexpressed ICOSL also expressed higher ICOS than those induced by HL-60 cells transduced with NC plasmids (Figure 3C). To further confirm the role of ICOSL as a Treg inducer, a neutralizing anti-ICOSL antibody was used to block the interaction of ICOS and ICOSL, and potently decreased the induction of CD25^+^Foxp3^+^ Tregs from CD4^+^ cells (Figure 3D). ICOS expression was also robustly reduced in these Tregs (Figure 3D). Additionally, co-culture of HEL cells resulted in the inhibition of Th1 cytokine profile (decreased IFN-γ) and promoting the expansion of Th17 cells from CD4^+^ cells (Figure S2).

### IL-10 secreted by TREG cells promotes the proliferation of AML cells

**Figure 4:**
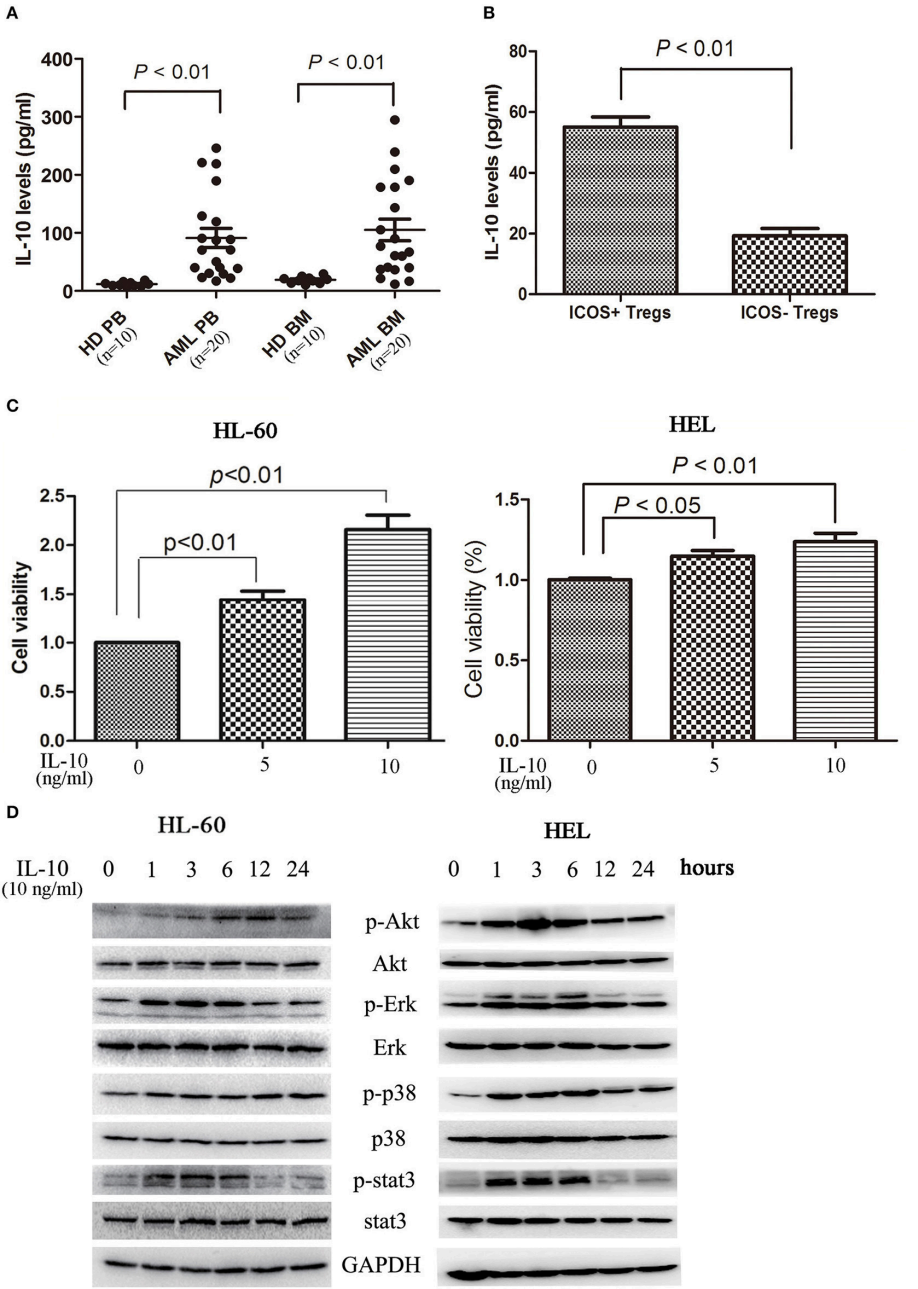
IL-10 promotes the proliferation of AML cells. **(A)** IL-10 levels were increased in plasma and bone marrow plasma in 20 AML patients compared with 10 healthy donors. Unpaired *t*-test was used to determine the differences. **(B)** The CD4^+^CD25^high^ICOS^+^ cells and CD4^+^CD25^high^ICOS^−^ cells were sorted and cultured in 200 μl X-VIVO™ 15 supplemented with 20 ng/ml IL-2 for 2 days, and the supernatants were subsequently collected and the levels of IL-10 were determined using a commercial ELISA kits. Data were expressed as mean ± SEM representing four independent experiments and unpaired *t*-test was used to determine the difference. **(C)** Treatment with IL-10 for 24 h promoted the proliferation of HL-60 cells and HEL cells in a dose-dependent manner. Data were expressed as mean ± SEM representing five independent experiments and ANOVA was used to determine the differences. **(D)** IL-10 promoted the phosphorylation of Akt, Erk1/2, p38, and Stat3. Images shown were representatives of three independent experiments.

In addition to direct killing of cytotoxic cells and modulation of other immune cells, Treg cells secrete immunomodulatory cytokines, in particular TGF-β and IL-10, to directly affect tumor cells. As shown in Figure 4A, elevated levels of IL-10 was observed in circulating blood and bone marrow microenvironment. As expected, ICOS^+^ Tregs secreted more IL-10 than ICOS^−^ Tregs (Figure 4B). IL-10 stimulation dose-dependently promoted the proliferation of HL-60 cells and HEL cells (Figure 4C). We further analyzed the signaling pathways closely related with cell proliferation. On IL-10 stimulation, Erk1/2, p38, and Stat3 were phosphorylated at 1 h, lasting for 5 h. Akt was also phosphorylated with the peak phosphorylation at 3–6 h apparently later than other three proteins in HL-60 cells and HEL cells (Figure 4D).

### Anti-ICOSL antibody restores C1498-induced elevation of regulatory T cells in an AML model

**Figure 5:**
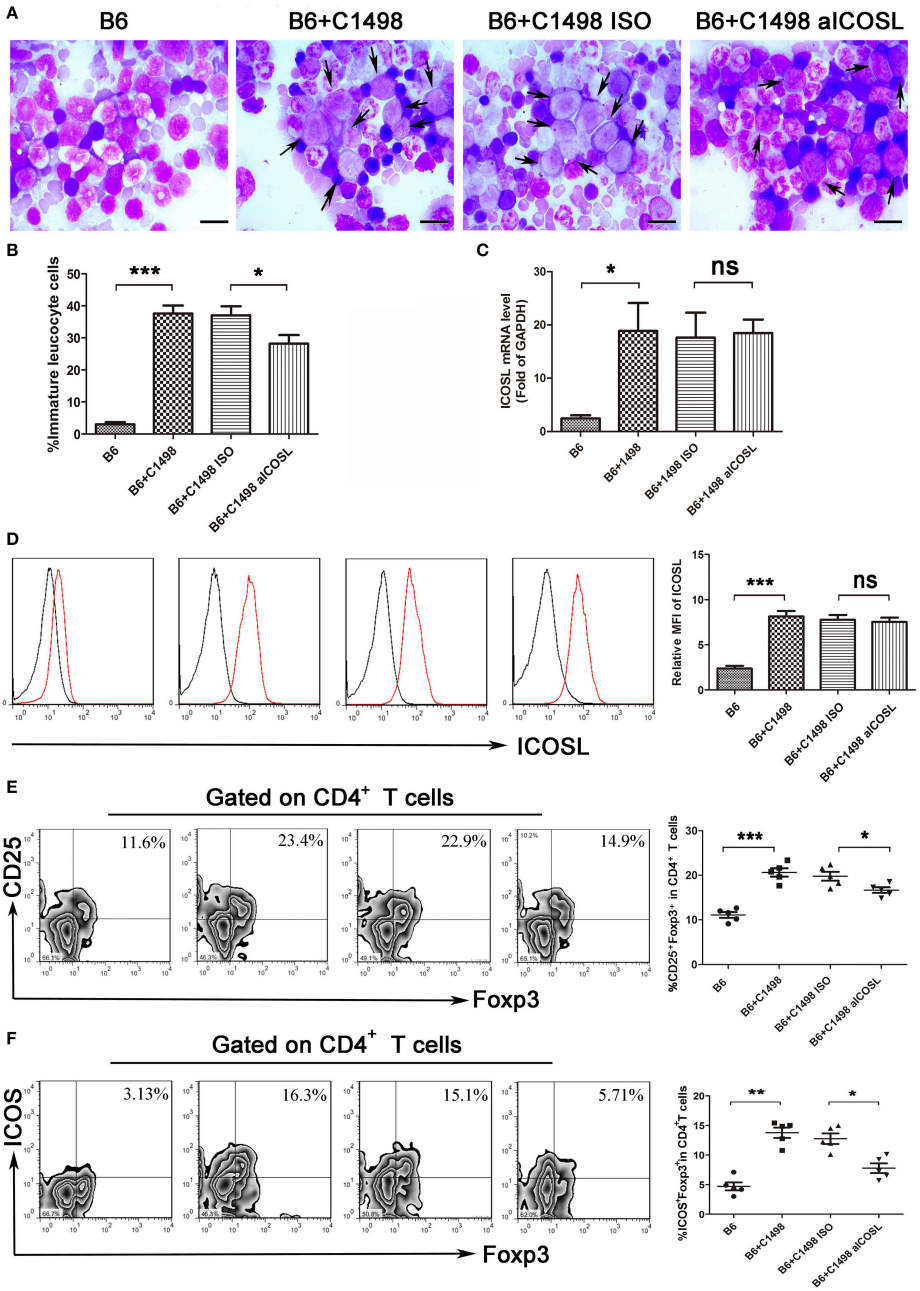
Blockade of ICOS signaling by anti-ICOSL mAb retards the progression of C1498-injected AML mice by impairing the generation of Tregs. **(A,B)** Representative images and statistical data about immature leucocyte cells were shown using Wright-stained bone marrow smears from 5 mice each group. The scale bars are10 μm, and arrows indicate immature leucocyte cells. ANOVA was used to determine the differences. **(C)** The mRNA expression of ICOSL of bone marrow mononuclear cells were determined using qRT-PCR in 5 mice each group. ANOVA was used to determine the differences. **(D)** Histograms showing the expression of ICOSL of bone marrow mononuclear cells were representatives of 5 mice each group. The black lines indicate isotype control, and the red lines indicate the expression of ICOSL. ANOVA was used to determine the differences. **(E)** The frequencies of CD4^+^CD25^+^Foxp3^+^ cells was significantly increased in the bone marrow microenvironment of C1498-injected mice and anti-ICOSL mAb dramatically reduced the generation of CD4^+^CD25^+^Foxp3^+^ cells in bone marrow. **(F)** Blockade of ICOS signaling by anti-ICOSL mAb reduced the generation of CD4^+^ICOS^+^Foxp3^+^ cells in bone marrow of C57B6/L mice elicited by injection of C1498 cells. Representative images and statistical data were shown for 5 mice each group. ANOVA was used to determine the differences. *P < 0.05, **P < 0.01, ***P < 0.001, NS stands for not significant.

The examinations of bone marrow smear and biopsy demonstrated that the C1498 AML model had been established on 15 days after intravenous injection (Figures 5A,B). We found the expression of ICOSL were gradually enhanced when C1498 cells were injected into the blood (Figure 1D). When the mice injected C1498 cells were sacrificed, the expression of ICOSL in mononuclear cells isolated from peripheral blood, bone marrow, and spleen were significantly increased at both mRNA and cell surface protein levels (Figures 5C,D, S3). The antibody against ICOSL did not affect the expression of ICOSL of mononuclear cells on these tissues (Figures 5C,D, Figure S3). The frequency of Treg cells and ICOS^+^ Tregs were significantly increased in peripheral blood, bone marrow and spleen in C1498 AML model compared to those of the vehicle mice (Figures 5E,F, Figure S3). Meanwhile, decreased Th1 cell frequency and increased Th17 and Th2 cell frequencies were also occurred in bone marrow and spleen in C1498 AML model compared to those of the vehicle mice (Figure S4). The antibody against ICOSL markedly decreased the frequency of Tregs and ICOS^+^ Tregs in peripheral blood, bone marrow and spleen (Figure 5E,F, Figure S3). Meanwhile, this antibody injected also increased the frequency of Th1 cells and decreased the frequencies of Th17 and Th2 in C1498 cells injected mice (Figure S3). Finally, the immature leucocyte cells in bone marrow smear were dramatically reduced in the mice injected the antibody against ICOSL (Figures 5A,B). These data demonstrate a crucial role of ICOS/ICOSL signaling for the generation of ICOS^+^ Tregs.

### Prognostic significance of the ICOSL expression of patient AML cells, TREGs, and ICOS^+^ TREGs

**Figure 6:**
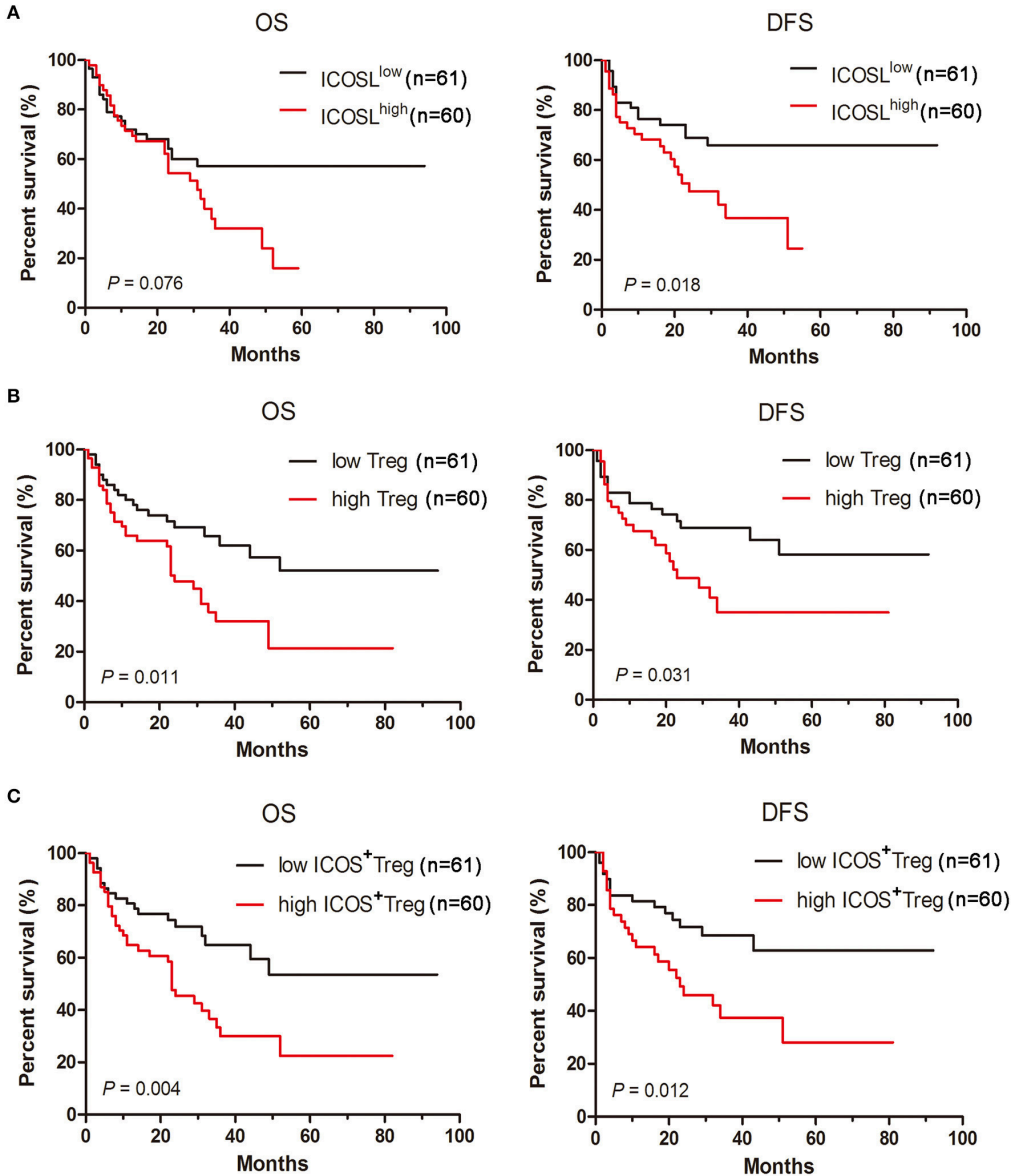
Increased expression of ICOSL in patient AML cells, increased frequencies of Tregs and ICOS^+^ Tregs predict poor survival in AML patients. Kaplan-Meier curves for overall survival and disease-free survival were assessed in ICOSL expression of patient AML cells **(A)**, frequencies of Tregs **(B)** and ICOS^+^ Tregs **(C)**, in bone marrow microenvironment of 121 patients with AML. The log-rank method was used to test for differences in survival.

To investigate whether the ICOS/ICOSL pathway affects the clinical outcome, the survival of AML patients was examined. When AML patients were classified into two groups using the median of ICOSL positivity, cases in high ICOSL group (*n* = 61, named ICOSL^high^ AML) showed a short but not statistically significant overall survival and a markedly shorter disease-free survival compared with ICOSL^low^ AML cases (*n* = 60; Figure 6A). Meanwhile, the influence of Treg cell frequency in bone marrow on patient survival was analyzed. The patients were divided into two groups based on the median frequency of Treg cells. The overall survival and disease-free survival in high Treg group were significantly shorter than those in low Treg group (Figure 6B), suggesting that increased Treg cell frequency might be an unfavorable prognostic marker for AML patients. Furthermore, the frequency of ICOS^+^Tregs was determined and the patients were divided into two groups based on the median frequency of ICOS^+^Tregs. The overall survival and disease-free survival in high ICOS^+^Treg group was evidently shorter than those in low ICOS^+^Treg group (Figure 6C).

## Discussion

Costimulatory molecules of the B7 family are essential for T cell activation during antigen recognition on APCs and other tissues. In tumor, some B7 family members are immune suppressive and facilitate immune evasion. For example, B7-H1 broadly detectable on the majority of tumors, can deliver an potent inhibitory signal to its receptor PD-1 on T cells, whereby B7-H1 inhibits the expansion and survival of antitumor T cells and is associated with worse prognosis (26). The results presented in this study demonstrate that a high proportion of AML cells express cell surface ICOSL. We found that a proinflammatory cytokine TNF-α could upregulate the expression of ICOSL in AML cells. Blockade of ICOS signaling by application of a blocking anti-ICOSL mAb impaired the interaction between Treg cells and tumor cells and improved disease.

ICOSL expression in the tumor microenvironment serves as a potential source mediating powerful costimulation for the tumor-infiltrating lymphocytes. For example, ICOSL expressed on mesenchymal stem cells, and melanoma cells, can promote the induction and expansion of IL-10-producing CD4^+^ T cells (12, 27). In this study, we found that ICOSL expressed by AML cells could costimulate Tregs to induce high levels of Foxp3, CD25, and ICOS. The co-culture of CD4^+^ T cells and AML cells suggest that tumor cells themselves may act as direct APC. In contrast, blockade of ICOSL during T cell activation reduced Foxp3 expression but did not eliminate it, indicating that there are some other factor influencing Foxp3 expression, such as TCR signals and perhaps other tumor factors. Thus, our results are consistent with a previous report that signaling via ICOS considerably contributes to the survival and expansion of Foxp3^+^ Tregs and does not only control the pool size of effector and memory T cells (28). Several studies have also shown that AML cells expressed other molecules, such as indoleamine 2, 3-dioxygenase (29, 30), to induce the expansion of Tregs. Additionally, B7-2 expressed by AML cells has also been reported to be capable to directly provoking Th-cell responses, and subsequently, the interaction between stimulated T cells and AML cells results in upregulation of inhibitory B7-H1 and B7-DC on AML cells that may contribute to immune evasion in AML (19).

As found here, increased expression of ICOS has been found in subsets of activated Foxp3^+^ Tregs from peripheral blood (31). Since responses measured in the blood may not always overlap with those measured in the bone marrow, it is critical to identify the relation between immune cells and signaling at the tumor microenvironment. Elevated ICOS^+^ Tregs were also present in bone marrow of AML patients, indicating an active presence of these cells in the tumor microenvironment. Moreover, ICOS^+^ Tregs were demonstrated to elicit superior suppressive activity in our and other studies (32). Taken together, these data indicate that ICOS^+^ Tregs are a subset of recently activated Tregs as a result of contact with tumor self-antigens and are critical in maintaining self-tolerance. This is in keeping with recent data showing an association of ICOS expression and IL-10-producing capacity in human Tregs and Tr1 cells. Data showing reduced ICOS^+^ Treg frequencies in the pancreatic tissue in a mouse model of type I diabetes further support this notion (33). ICOS^+^Tregs confer a strong suppressive capacity to influence other anti-tumor immune effector cells and have be a stronger predictor for clinical outcome than total Tregs in several tumors (34). It will be important to determine the levels of ICOSL expression in a larger number of primary AML samples and determine whether ICOSL^+^ tumor cells are associated with increased infiltrating Tregs and poorer overall survival. In this study, we found that AML patients with the high expression of ICOSL have a shorter disease-free survival than those with the low expression of ICOSL. Moreover, the frequency of ICOS^+^ Tregs was a better predictor than that of total Tregs in patients with AML.

From a clinical standpoint, inhibition of ICOS expression or blocking ICOS costimulatory signaling may be of therapeutic benefit. In this study, we found that blockade of ICOSL *in vivo* can in fact reduced Tregs in the tumor environment; however, careful dissection of the role of ICOS costimulation blockade on Tregs vs. effector T cells needs to be addressed. Furthermore, ICOSL on AML cells enhanced Treg proliferation and stimulated them to produce soluble factors, one of which, IL-10, further promotes the proliferation of AML cells.

In summary, our results suggested that AML cells expressed ICOSL promote the expansion of ICOS^+^ Tregs in tumor environment, and ICOS^+^ Tregs further promote the proliferation of AML cells through secreting IL-10. Therefore, we speculate that blockade of ICOS/ICOSL signaling may be a specific, targeted therapy for AML.

## Author contributions

SZ and KY designed the study and supervised the manuscript preparation. YH and YD performed the research. YH, YD, and SZ analyzed the data and wrote the manuscript. QY, WX, SJ, and ZY participated in the collection of patients' data. All of the authors agreed to submit the final manuscript.

### Conflict of interest statement

The authors declare that the research was conducted in the absence of any commercial or financial relationships that could be construed as a potential conflict of interest.

## Supplementary material

Table S1 Clinical characteristics of patients with AML excluding APL with *t*(15;17)(q22;q12); PML-RARA.

Table S2 The sequences of the primers used for real-time qPCR.

Figure S1 Several other cytokines including IFN-γ, IL-10, IL-17A, or IL-21 almost do not influence the expression of ICOSL on two AML cell lines HL-60 and HEL.

Figure S2 Interaction with HEL cells suppresses Th1 cells and promotes Th17 cells in the CD4^+^ T cells.

Figure S3 Blockade of ICOS signaling by anti-ICOSL mAb increases Th1 cells and decreases Th17 and Th2 cells in C1498-injected AML mice.

Figure S4 Blockade of ICOS signaling by anti-ICOSL mAb impairs the generation of Tregs in peripheral blood and spleen of C1498-injected mice.
