# Supplementary material for: Acute Myeloid Leukemia Cells Express ICOS Ligand to Promote the Expansion of Regulatory T Cells
Source: Front Immunol. 2018 Sep 26;9:2227. doi: 10.3389/fimmu.2018.02227 (PMC6168677; doi:10.3389/fimmu.2018.02227)
Supplement: Table S1 — Clinical characteristics of patients with AML excluding APL with t(15;17)(q22;q12); PML-RARA. [file Data_Sheet_1.docx]

**Table S1** Clinical characteristics of patients with AML excluding APL with t(15;17)(q22;q12); PML-RARA.

| **Patient no** | **Sex** | **Age,y** | **Histologic diagnosis** | **Included date** |
| --- | --- | --- | --- | --- |
| 1 | M | 43 | Acute myelomonocytic leukemia | 2009/2/25 |
| 2 | M | 26 | AML with t(8;21)(q22;q22); RUNX1-RUNX1T1 | 2009/2/25 |
| 3 | M | 36 | Acute erythroid leukemia | 2009/5/18 |
| 4 | F | 51 | Acute myelomonocytic leukemia | 2009/9/14 |
| 5 | F | 46 | Acute monoblastic/monocytic leukemia | 2009/11/23 |
| 6 | M | 26 | Acute monoblastic/monocytic leukemia | 2009/11/30 |
| 7 | M | 46 | Acute myelomonocytic leukemia | 2010/1/5 |
| 8 | M | 22 | Acute monoblastic/monocytic leukemia | 2010/2/9 |
| 9 | M | 40 | Acute erythroid leukemia | 2010/5/13 |
| 10 | M | 40 | AML with maturation | 2010/7/26 |
| 11 | F | 40 | Acute myelomonocytic leukemia | 2010/8/10 |
| 12 | M | 17 | AML with t(8;21)(q22;q22); RUNX1-RUNX1T1 | 2010/8/11 |
| 13 | M | 29 | Acute myelomonocytic leukemia | 2010/9/11 |
| 14 | M | 33 | Acute myelomonocytic leukemia | 2010/9/13 |
| 15 | M | 38 | Acute myelomonocytic leukemia | 2010/10/21 |
| 16 | F | 36 | Acute myelomonocytic leukemia | 2010/10/28 |
| 17 | F | 59 | Acute monoblastic/monocytic leukemia | 2010/11/10 |
| 18 | M | 22 | Acute monoblastic/monocytic leukemia | 2010/12/21 |
| 19 | F | 23 | AML with t(8;21)(q22;q22); RUNX1-RUNX1T1 | 2011/1/31 |
| 20 | M | 41 | Acute myelomonocytic leukemia | 2011/2/11 |
| 21 | M | 33 | AML with t(16;16)(p13.1;q22); CBFB-MYH11 | 2011/5/25 |
| 22 | F | 58 | Acute monoblastic/monocytic leukemia | 2011/6/28 |
| 23 | F | 21 | Acute myelomonocytic leukemia | 2011/7/27 |
| 24 | M | 49 | Acute monoblastic/monocytic leukemia | 2011/9/19 |
| 25 | M | 32 | Acute myelomonocytic leukemia | 2011/10/18 |
| 26 | F | 42 | Acute myelomonocytic leukemia | 2011/11/30 |
| 27 | F | 38 | Acute monoblastic/monocytic leukemia | 2011/12/4 |
| 28 | M | 34 | AML with t(8;21)(q22;q22); RUNX1-RUNX1T1 | 2011/12/5 |
| 29 | F | 54 | Acute monoblastic/monocytic leukemia | 2012/1/31 |
| 30 | M | 58 | Acute myelomonocytic leukemia | 2012/3/5 |
| 31 | F | 56 | AML with t(8;21)(q22;q22); RUNX1-RUNX1T1 | 2012/3/6 |
| 32 | M | 36 | AML with t(16;16)(p13.1;q22); CBFB-MYH11 | 2012/4/13 |
| 33 | F | 60 | Acute myelomonocytic leukemia | 2012/4/25 |
| 34 | M | 32 | Acute myelomonocytic leukemia | 2012/7/4 |
| 35 | F | 55 | Acute myelomonocytic leukemia | 2012/8/10 |
| 36 | M | 57 | AML with mutated CEBPA | 2012/8/23 |
| 37 | F | 35 | Acute monoblastic/monocytic leukemia | 2012/8/23 |
| 38 | M | 55 | Acute erythroid leukemia | 2012/10/15 |
| 39 | F | 58 | AML with maturation | 2012/11/10 |
| 40 | M | 49 | AML with t(8;21)(q22;q22); RUNX1-RUNX1T1 | 2012/12/28 |
| 41 | F | 26 | Acute monoblastic/monocytic leukemia | 2013/1/16 |
| 42 | M | 31 | AML with maturation | 2013/3/6 |
| 43 | M | 54 | AML with maturation | 2013/3/13 |
| 44 | M | 46 | Acute myelomonocytic leukemia | 2013/3/20 |
| 45 | F | 24 | Acute monoblastic/monocytic leukemia | 2013/3/30 |
| 46 | F | 23 | AML with t(8;21)(q22;q22); RUNX1-RUNX1T1 | 2013/4/18 |
| 47 | M | 18 | Acute megakaryoblastic leukemia | 2013/4/22 |
| 48 | F | 39 | Acute myelomonocytic leukemia | 2013/4/24 |
| 49 | F | 56 | Acute monoblastic/monocytic leukemia | 2013/4/29 |
| 50 | M | 29 | Acute myelomonocytic leukemia | 2013/5/14 |
| 51 | M | 36 | Acute myelomonocytic leukemia | 2013/5/22 |
| 52 | M | 26 | AML with maturation | 2013/6/14 |
| 53 | M | 46 | Acute monoblastic/monocytic leukemia | 2013/7/2 |
| 54 | M | 50 | Acute erythroid leukemia | 2013/7/4 |
| 55 | M | 17 | AML with t(8;21)(q22;q22); RUNX1-RUNX1T1 | 2013/7/19 |
| 56 | F | 56 | Acute monoblastic/monocytic leukemia | 2013/7/23 |
| 57 | M | 38 | Acute monoblastic/monocytic leukemia | 2013/8/6 |
| 58 | F | 28 | Acute monoblastic/monocytic leukemia | 2013/8/9 |
| 59 | M | 50 | Acute monoblastic/monocytic leukemia | 2013/8/27 |
| 60 | M | 46 | AML with mutated NPM1 | 2013/10/7 |
| 61 | F | 20 | Acute monoblastic/monocytic leukemia | 2013/10/14 |
| 62 | F | 18 | AML with t(8;21)(q22;q22); RUNX1-RUNX1T1 | 2013/10/25 |
| 63 | F | 28 | Acute myelomonocytic leukemia | 2013/11/4 |
| 64 | F | 36 | Acute myelomonocytic leukemia | 2013/11/6 |
| 65 | F | 28 | Acute monoblastic/monocytic leukemia | 2013/11/26 |
| 66 | F | 50 | AML with t(8;21)(q22;q22); RUNX1-RUNX1T1 | 2013/12/2 |
| 67 | M | 59 | Acute myelomonocytic leukemia | 2013/12/13 |
| 68 | M | 23 | Acute myelomonocytic leukemia | 2013/12/19 |
| 69 | F | 50 | Acute myelomonocytic leukemia | 2013/12/19 |
| 70 | F | 57 | Acute myelomonocytic leukemia | 2014/1/1 |
| 71 | M | 44 | Acute monoblastic/monocytic leukemia | 2014/1/21 |
| 72 | M | 56 | Acute monoblastic/monocytic leukemia | 2014/3/3 |
| 73 | M | 55 | Acute monoblastic/monocytic leukemia | 2014/4/1 |
| 74 | F | 55 | Acute myelomonocytic leukemia | 2014/4/18 |
| 75 | F | 40 | Acute monoblastic/monocytic leukemia | 2014/4/18 |
| 76 | F | 32 | Acute monoblastic/monocytic leukemia | 2014/4/30 |
| 77 | M | 57 | Acute monoblastic/monocytic leukemia | 2014/5/4 |
| 78 | F | 25 | Acute monoblastic/monocytic leukemia | 2014/5/4 |
| 79 | M | 51 | Acute monoblastic/monocytic leukemia | 2014/5/22 |
| 80 | M | 55 | Acute myelomonocytic leukemia | 2014/5/23 |
| 81 | M | 37 | Acute monoblastic/monocytic leukemia | 2014/5/24 |
| 82 | F | 25 | Acute myelomonocytic leukemia | 2014/6/16 |
| 83 | M | 54 | Acute myelomonocytic leukemia | 2014/6/18 |
| 84 | M | 23 | AML with maturation | 2014/6/27 |
| 85 | F | 44 | Acute monoblastic/monocytic leukemia | 2014/6/30 |
| 86 | F | 23 | Acute monoblastic/monocytic leukemia | 2014/7/1 |
| 87 | M | 22 | Acute monoblastic/monocytic leukemia | 2014/7/8 |
| 88 | M | 58 | Acute myelomonocytic leukemia | 2014/7/18 |
| 89 | F | 34 | Acute myelomonocytic leukemia | 2014/7/25 |
| 90 | M | 40 | Acute monoblastic/monocytic leukemia | 2014/7/30 |
| 91 | M | 37 | Acute myelomonocytic leukemia | 2014/8/1 |
| 92 | F | 59 | Acute monoblastic/monocytic leukemia | 2014/8/14 |
| 93 | M | 39 | AML with t(8;21)(q22;q22); RUNX1-RUNX1T1 | 2014/8/29 |
| 94 | F | 30 | Acute myelomonocytic leukemia | 2014/10/23 |
| 95 | M | 49 | AML with t(8;21)(q22;q22); RUNX1-RUNX1T1 | 2014/11/4 |
| 96 | F | 18 | Acute monoblastic/monocytic leukemia | 2014/12/2 |
| 97 | F | 48 | AML with t(8;21)(q22;q22); RUNX1-RUNX1T1 | 2014/12/11 |
| 98 | F | 37 | Acute monoblastic/monocytic leukemia | 2014/12/23 |
| 99 | M | 48 | Acute erythroid leukemia | 2014/12/24 |
| 100 | M | 34 | Acute myelomonocytic leukemia | 2014/12/26 |
| 101 | M | 29 | Acute erythroid leukemia | 2015/1/19 |
| 102 | M | 45 | Acute monoblastic/monocytic leukemia | 2015/1/20 |
| 103 | M | 20 | AML with t(16;16)(p13.1;q22); CBFB-MYH11 | 2015/1/26 |
| 104 | M | 23 | Acute monoblastic/monocytic leukemia | 2015/3/13 |
| 105 | M | 17 | AML with t(8;21)(q22;q22); RUNX1-RUNX1T1 | 2015/3/17 |
| 106 | M | 37 | Acute myelomonocytic leukemia | 2015/3/19 |
| 107 | F | 56 | Acute myelomonocytic leukemia | 2015/4/7 |
| 108 | F | 46 | Acute myelomonocytic leukemia | 2015/4/8 |
| 109 | M | 49 | Acute monoblastic/monocytic leukemia | 2015/4/24 |
| 110 | F | 25 | Acute monoblastic/monocytic leukemia | 2015/6/2 |
| 111 | M | 52 | AML with maturation | 2015/7/10 |
| 112 | M | 60 | AML with maturation | 2015/7/14 |
| 113 | F | 39 | Acute monoblastic/monocytic leukemia | 2015/8/27 |
| 114 | F | 37 | Acute myelomonocytic leukemia | 2015/9/17 |
| 115 | M | 24 | AML with t(16;16)(p13.1;q22); CBFB-MYH11 | 2015/9/24 |
| 116 | M | 46 | Acute myelomonocytic leukemia | 2015/10/30 |
| 117 | M | 39 | AML with mutated CEBPA | 2015/11/2 |
| 118 | F | 55 | AML with maturation | 2015/11/2 |
| 119 | M | 47 | AML with maturation | 2015/11/10 |
| 120 | F | 41 | AML with maturation | 2015/11/19 |
| 121 | F | 55 | Acute monoblastic/monocytic leukemia | 2015/12/7 |

**Table S2** The sequences of the primers used for real-time qPCR.

|  | Forward primer | Reverse primer |
| --- | --- | --- |
| human ICOSL | 5’-CCCAGGACGAGCAGAAGTTT-3’ | 5’-TGAAGTTTGCTGCCACATGC-3’ |
| mouse ICOSL | 5’-TGGAAGAGGTGGTCAGGC-3’ | 5’-TTAGGCTATTGTCCGTTGTG-3’ |
| human GAPDH | 5’-ATCATCAGCAATGCCTCC-3’ | 5’-CATCACGCCACAGTTTCC-3’ |
| mouse GAPDH | 5’-ACCACAGTCCATGCCATCAC-3’ | 5’-TCCACCACCCTGTTGCTGTA-3’ |


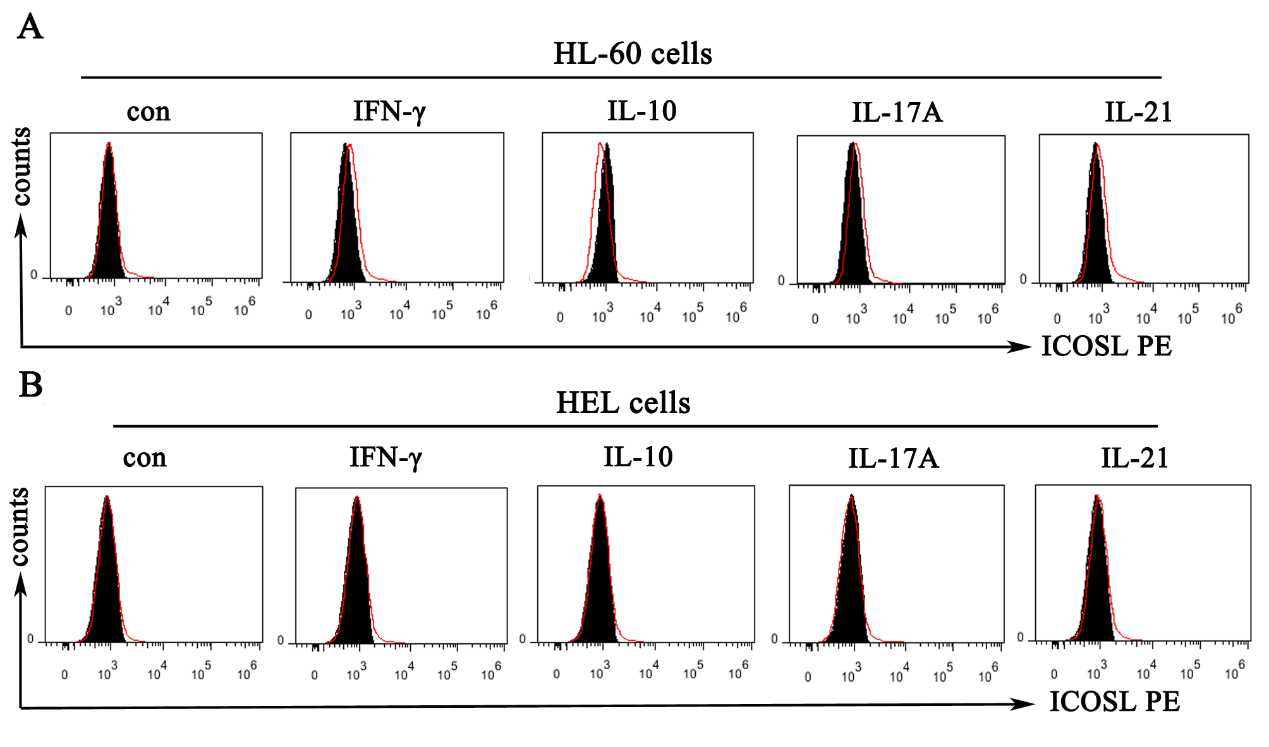


**FIGURE S1** Several other cytokines including IFN-γ, IL-10, IL-17A, or IL-21 almost do not influence the expression of ICOSL on two AML cell lines HL-60 and HEL. HL-60 cells **(A)** and HEL cells **(B)** were treated with 1 µg/ml IFN-γ, 10 ng/ml IL-10, 200 ng/ml IL-17A, or 200 ng/ml IL-21 for 48 hours, and the expression of ICOSL was subsequently determined using a flow cytometry-based assay. Overlay histograms showing antibody stains (solid red) and isotype stains (filled black) were representatives of three independent experiments.


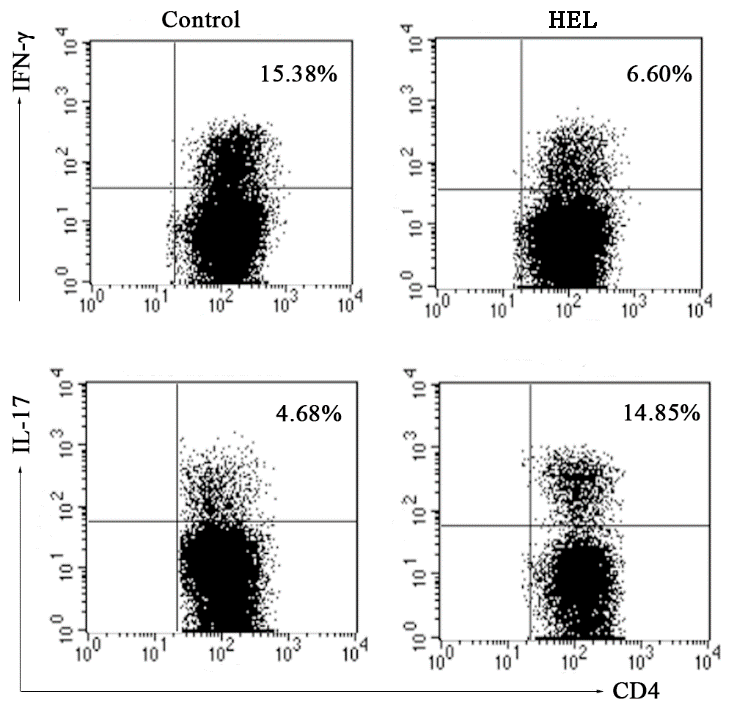


**FIGURE S2** Interaction with HEL cells suppresses Th1 cells and promotes Th17 cells in the CD4^+^ T cells. CD4^+^ T cells were incubated with or without HEL cells for 48 hours in the presence of 20 ng/ml IL-2, and in the last 5 hours, the cells were stimulated with PMA and ionomycin in the presence of brefeldin A. Then, the cells were collected, and stained for surface CD4 and intracellular IFN-γ and IL-17A, and determined on a flow cytometry. Images shown were representatives of three independent experiments.


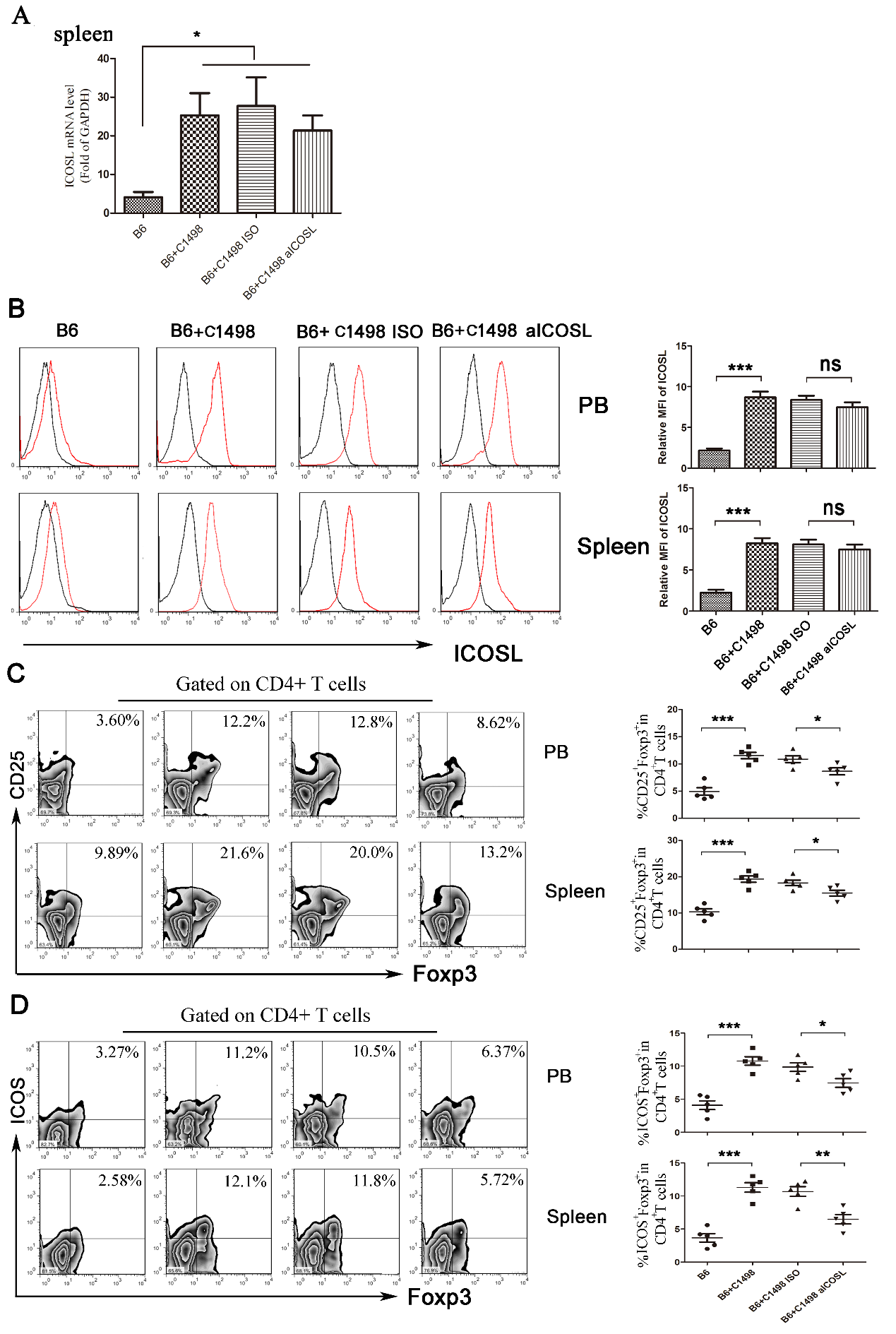


**FIGURE S3** Blockade of ICOS signaling by anti-ICOSL mAb impairs the generation of Tregs in peripheral blood and spleen of C1498-injected mice. **(A)** The mRNA expression of ICOSL of spleen cells were determined using qRT-PCR in 5 mice each group. ANOVA was used to determine the differences. **(B)** Histograms showing the expression of ICOSL of peripheral blood mononuclear cells and spleen cells were representatives of 5 mice each group. The black lines indicate isotype control, and the red lines indicate the expression of ICOSL. ANOVA was used to determine the differences. **(C & D)** The frequencies of CD4^+^CD25^+^Foxp3^+^ cells and CD4^+^ICOS^+^Foxp3^+^ cells were significantly increased in the peripheral blood and spleen of C1498-injected mice and anti-ICOSL mAb dramatically reduced the generation of CD4^+^CD25^+^Foxp3^+^ cells and CD4^+^ICOS^+^Foxp3^+^ cells in peripheral blood and spleen. Representative images and statistical data were shown for 5 mice each group. ANOVA was used to determine the differences. ^*^*P* < 0.05, ^***^*P* < 0.001, NS stands for not significant.





**FIGURE S4** Blockade of ICOS signaling by anti-ICOSL mAb increases Th1 cells and decreases Th17 and Th2 cells in C1498-injected AML mice. After treatment with anti-ICOSL mAb, the frequencies of Th1, Th17 and Th2 cells in spleen and bone marrow were determined using a flow-cytometry-based assay. Representative images (left panel) and statistical data (right panel) about IFN-γ-producing (Th1) cells **(A)**, IL-17A-producing (Th17) cells **(B)**, IL-4-producing (Th2) cells **(C)**, and IL-10-producing (Th2) cells **(D)** were showed for 5 mice each group. ANOVA was used to determine the differences. ^*^*P* < 0.05, ^***^*P* < 0.001, NS stands for not significant.
